# Effective surgical management of glomus tympanicum tumor using diode laser: A case report study^☆^

**DOI:** 10.1016/j.ijscr.2023.108356

**Published:** 2023-05-25

**Authors:** Ahmad Alkheder, Amjad Ghareeb, Mohammad Sadek Almasalmeh, Abdulmajeed Yousfan

**Affiliations:** Department of Otorhinolaryngology, Al Mouwasat University Hospital, Faculty of Medicine, Damascus University, Damascus, Syria

**Keywords:** Glomus tympanicum, Paraganglioma, Middle ear, Benign tumor, Diode laser

## Abstract

**Introduction:**

Glomus tympanicum is an extremely rare benign paraganglioma of the middle ear. The distinctive features of these tumors include their propensity for recurrence following treatment and their remarkably vascular nature, posing significant challenges to surgeons and necessitating the development of effective surgical techniques.

**Case presentation:**

A 56-year-old female presented with pulsatile tinnitus persisting for a year. Examination revealed a pulsating red mass in the lower section of the tympanic membrane. Computed tomography confirmed the presence of a mass occupying the middle ear, which was diagnosed as a glomus tympanicum tumor. The patient underwent surgical excision of the tumor, followed by diode laser application for coagulation at the site of the tumor. Histopathological examination confirmed the clinical diagnosis.

**Discussion:**

Glomus tympanicum tumors are rare neoplasms that arise in the middle ear. The surgical management of these tumors varies depending on the size and extent of the lesion. Various techniques are available for excision, including bipolar cautery and laser. Laser has emerged as an effective method for reducing tumor mass and controlling intraoperative bleeding, with positive indications after surgery.

**Conclusion:**

Based on our case report, laser can be considered an effective and safe method for excision of glomus tympanicum, with positive indications for controlling intraoperative bleeding and reducing tumor mass.

## Highlights

• Glomus tympanicum is a rare, neuroendocrine, highly vascular benign tumor of the middle ear.
• Surgical resection is the treatment of choice for glomus tympanicum tumors.
• Diode laser was applied to the vascular supply of the tumor, as well as to the site and mass of the tumor.
• This method effectively controls bleeding and reduces the highly vascularized tumor mass.
• Combining laser and surgery yields improved outcomes and greater maneuverability.

## 1 Introduction

Glomus tympanicum is a rare, highly vascularized, benign neuroendocrine tumor that belongs to the group of glomus tumors, also known as paragangliomas, which can occur in various parts of the body, including the carotid bodies. It is the most common primary neoplasm of the middle ear and the second most common tumor of the temporal bone. Due to its highly vascular nature, glomus tympanicum poses a challenge during surgical intervention and has a tendency to recur after treatment, with a recurrence rate of 17.1 % after 10 years [1], [2], [3].

In this report, we present a case of glomus tympanicum tumor resection using a postauricular approach and a diode laser. This work is also reported in line with SCARE criteria which helped to improve the transparency and quality of this case report [4].

## 2 Case presentation

A 56-year-old female was admitted to the hospital due to low-frequency pulsating tinnitus in her right ear that had been present for a year and was continuous. The tinnitus was not alleviated by noise. She also reported constant moderate hearing loss in her right ear for the past year and intermittent right ear pain without any history of ear discharge or other ear symptoms. There was no history of acoustic or mechanical trauma. The patient had a history of tension headaches, which were relieved by analgesics. No symptoms related to the throat, larynx, or nose were reported.

**Fig. 1:**
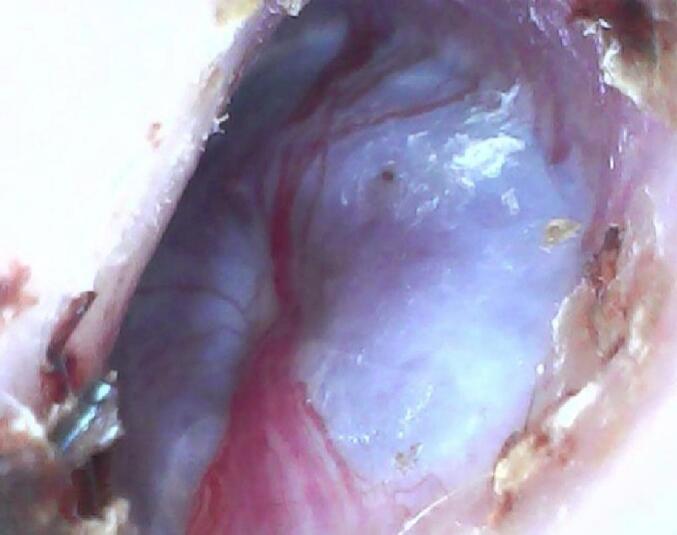
A reddish mass is seen in the right ear, causing bulging in the lower part of the tympanic membrane.

**Fig. 2:**
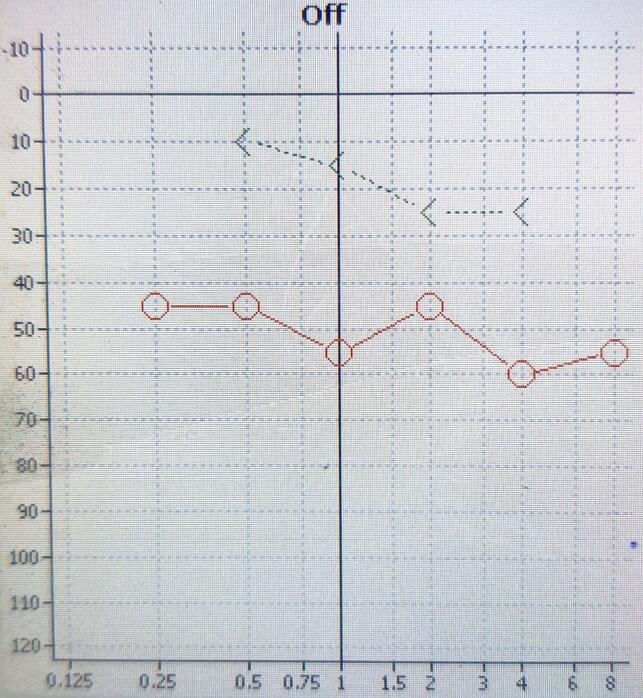
Audiogram showing conductive hearing loss in the right ear.

**Fig. 3:**
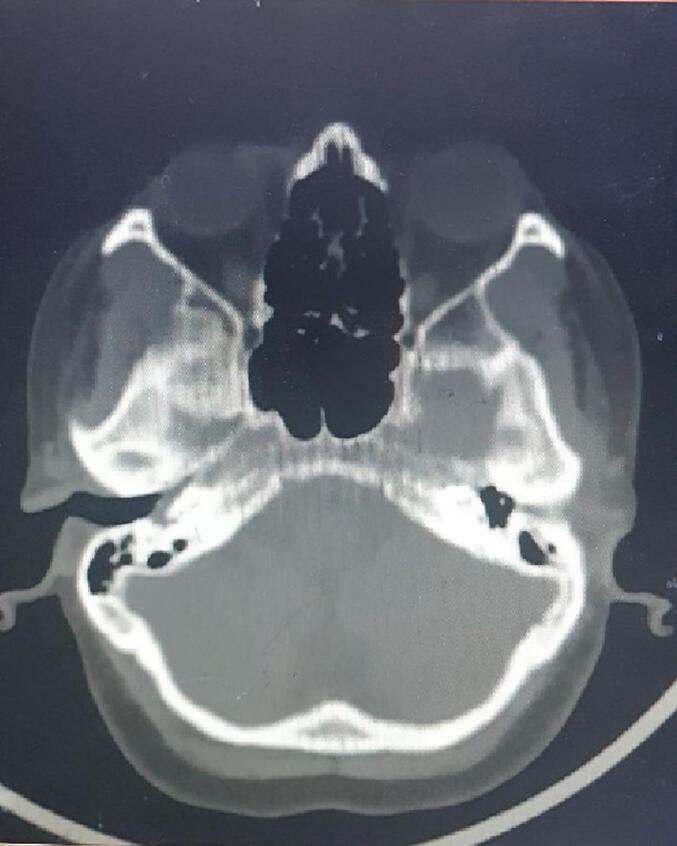
High-resolution computed tomography scan showing a mass on the promontory and occupying the right middle ear.

Upon clinical examination, a reddish mass was observed in the right ear, causing a bulge in the lower part of the tympanic membrane that was clearly pulsatile (Fig. 1). The left ear, nose, and throat appeared normal. Pure tone audiometry revealed a conductive hearing loss pattern in the right ear (Fig. 2). A high-resolution computerized tomography scan (HRCT scan) of the right temporal bone revealed a soft tissue density medial to the tympanic membrane, occupying the right middle ear. No obvious bony damage was observed, and the ossicles were intact and enhanced the contrast material after injection (Fig. 3). The facial nerve canal, external ear canal, cochlea, and semicircular canals were also found to be intact.

**Fig. 4:**
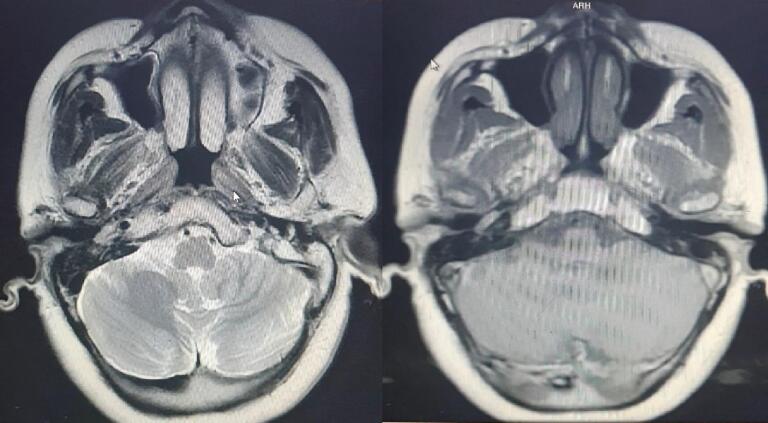
A MRI showing a lesion nearness the cochlea with high signal on T2, clear borders and good enhance.

An MRI was performed, which revealed a lesion measuring 5 × 13 mm near the cochlea with a high signal on T2 and clear borders and good enhancement after deleting the fat (Fig. 4). The tumor was classified as type 1 using the Glasscock–Jackson classification [5], class A using the Fisch classification [6], and class A2 using the modified Fisch and Mattox classification [7], based on the extent of the tumor shown by radiography.

**Fig. 5:**
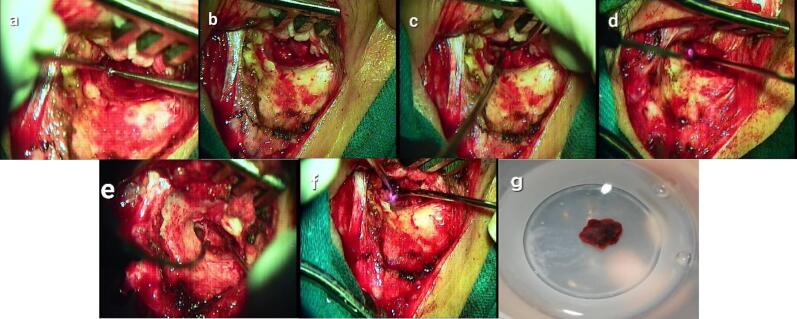
(a) Atticotomy performed using a microcurrette. (b) the tumor (indicated by the star). (c) Dissection of the tumor on the Malleus and Incus, tympanic sinus and eustachian tube region. (d) Cauterize of the tumor using a diode laser. (e) The promontory is exposed after dissecting a part of the mucous membrane, revealing the origin of the tumor. (f) Coagulation of the second vessel supplying the tumor, affecting the lower parts of the promontory. (g) Complete removal of the tumor from the middle ear.

Surgical resection was performed, beginning with the stages of Myringoplasty with a graft from the fascia of the temporal muscle, and retroauricular incision established access to the tympanic membrane. After reaching the middle ear, a simple atticotomy was performed using a microcurette (Fig. 5a), followed by dissection of the tumor on Malleus and Incus, tympanic sinus, and eustachian tube region (Fig. 5c). The supplying vessel, one of the branches of the tympanic plexus on the promontory, was exposed, and the tumor was cauterized with a diode laser (Fig. 5d). A part of the mucosa was dissected on the promontory where the tumor originated, then the second supplying vessel was coagulated at the expense of the lower parts of the promontory (hypotympanum region) (Fig. 5f). The tumor was then dissected from the epitympanum, hypotympanum, and remnants adhered to the promontory, with excision of a portion of the promontory mucosa attached to the tumor. The place of origin of the tumor was cauterized with a diode laser using a power of 3 watts pulse mode. The tumor was removed from the middle ear cavity without bleeding (Fig. 5g), and gelfoam was placed in the middle ear. Myringoplasty was then performed to graft the damaged part of the tympanic membrane. The ossicles were completely preserved, and no cranial neuropathy occurred.

**Fig. 6:**
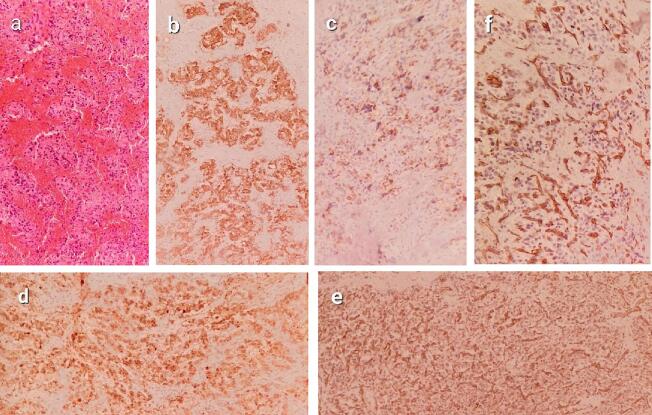
Histopathology. (a) Microscopic picture showing cells arranged in distinctive clusters, separated by fibrovascular stroma. Sustentacular cells wrap around chief cells, which are polygonal with abundant eosinophilic cytoplasm. (b) Strong diffuse positive cytoplasmic staining of synaptophysin in tympanic paraganglioma, which is neuroendocrine marker. (c) Strong diffuse granular positive staining of chromogranin A in chief cells of tympanic paraganglioma, confirming their neuroendocrine nature. (d) Strong diffuse positive cytoplasmic staining of S100 of chief cells of tympanic paraganglioma, which originate from the neural crest. (e) Strong diffuse positive cytoplasmic staining of SMA “smooth muscle actin” in sustentacular cells in tympanic paraganglioma, which wrap around chief cells. (f) Strong diffuse membranous positive staining of endothelial cells in vessels in the stroma of tympanic paraganglioma.

Histopathology (Fig. 6) Showed a tympanic paraganglioma (approximately 1.5 × 1 cm). The tumor was composed of chief cells arranged in distinct clusters and separated by fibrovascular stroma. Sustentacular cells surrounded the chief cells, which had a polygonal shape and abundant eosinophilic cytoplasm. Immunohistochemical staining of the tumor cells demonstrated positive expression of α-smooth muscle actin, synaptophysin, and chromogranin. Sustentacular cells were positive for S-100 protein, and there was positive staining of endothelial cells in the vessels within the stroma. Postoperative pure-tone audiometry revealed a normal hearing level, and the patient's right-sided pulsatile tinnitus completely resolved after surgery. There was no evidence of tumor recurrence observed during the two-year follow-up period.

## 3 Discussion

The retroauricular-transcanal approach is considered the optimal management for glomus tympanicum tumors that extend into the posterior mesotympanum (Class A2), as in the presented case. Studies have indicated that a stapedectomy-type transcanal approach can safely remove small glomus tympanicum tumors that are localized in the middle ear (Class A1), with visible margins and no tumor extension into the posterior mesotympanum. However, the retroauricular-transcanal approach and the modified procedure (glove finger flap technique), as performed in the presented case, offer a significant advantage by increasing the surgical field and allowing a wider exposure of the tumor through the ear canal. This facilitates better control of the ossicular chain and facial nerve, and the removal of glove finger skin flap enables better exposure and dissection of the windows' area and the facial nerve. Additionally, this approach facilitates the use of cautery during tumor dissection [7,8].

While microscopic ear surgeries are commonly performed for glomus tympanicum tumors, transcanal endoscopic ear surgeries are suitable for small tumors (modified Fisch and Mattox classification stage A1 tumors), but are not suitable for the presented case (A2). Also, we don't have the equipment of endoscopic ear surgery.

Controlling bleeding during surgery is a crucial issue for glomus tympanicum tumors, particularly in stage A2-B1 tumors, which can present a challenge. Coagulation offers several advantages, such as bleeding prevention and hemostasis to keep the surgical field clean, as well as reducing tumor volume. It is mandatory to locate the pedicle of the tumor and coagulate the vascular supply, as this can reduce the tumor volume and manage the extension of the mass [[7], [8], [9]].

In the presented case, a diode laser was used to shrink the tumor and coagulate the feeding vessels, as it is more effective in coagulation than traditional methods. The first study on the use of lasers in the excision of glomus tympanicum tumors was reported in 2004, which presented a series of nine patients who underwent successful resection of Type A glomus tympanicum tumors with the use of laser coagulation [10].

Laser excision of glomus tympanicum tumors (Type A) provides good exposure with clear operating fields, minimal bleeding, and excellent surgical control. Good anatomical understanding combined with experience in using lasers ensures effective treatment of glomus tympanicum tumors with no complications and good long-term results. The advantage of laser is its capacity to minimize blood loss through progressive coagulation and shrinkage of the tumor volume. This allows for good visualization of the surrounding structures with minimal mechanical trauma and complete excision of the tumor with coagulation of the residual feeding vessel. This is facilitated with a concomitant reduction in the risk of recurrence. The diode laser emits laser light at a wavelength of 810 nm, which provides deeper penetration than the KTP laser and is similar to the Nd-YAG laser, offering efficient coagulation of blood vessels, similar to the KTP laser [5].

## 4 Conclusion

Glomus tympanicum tumors are rare, benign neoplasms that occur in the middle ear and are highly vascularized. Our experience suggests that the use of diode laser in the surgical management of glomus tympanicum tumors is a safe and effective alternative that may offer advantages over conventional surgical methods. However, further research is needed to evaluate the long-term outcomes and results of diode laser excision in the treatment of glomus tympanicum tumors.

## Ethical approval

Ethical approval was taken from the University's Deanship.

## Funding

N/A. We received no funding in any form.

## Sources of funding

This research did not receive any specific grant from funding agencies in the public, commercial, or not-for-profit sectors.

## Guarantor

Ahmad Alkheder.

## Registration of research studies

This case report is not a first time of reporting, new device or surgical technique. So I would not need a Research Registry Unique identifying number (UIN).

## Consent of patient

Patient's parent consent was taken for publishing this case and the images. A copy of the written consent is available for review by the Editor-in-Chief of this journal on request.

## Provenance and peer review

Not commissioned, externally peer-reviewed.

## Availability of data and materials

All data are available from the corresponding author on reasonable request.

The case has not been presented at a conference or regional meeting.

## CRediT authorship contribution statement

**Ahmad Alkheder:** Validation, Writing – review & editing, Visualization, Methodology, Software, Writing – original draft, Formal analysis. **Amjad Ghareeb:** Validation, Writing – review & editing, Visualization, Methodology, Software, Writing – original draft, Formal analysis. **Mohammad Sadek Almasalmeh:** Validation, Formal analysis, Writing – review & editing. **Abdulmajeed Yousfan:** Supervision, Writing – review & editing, Project administration.

## Declaration of competing interest

The Authors disclose no conflicts.
